# B7-H3 Promotes Pathogenesis of Autoimmune Disease and Inflammation by Regulating the Activity of Different T Cell Subsets

**DOI:** 10.1371/journal.pone.0130126

**Published:** 2015-06-11

**Authors:** Liqun Luo, Gefeng Zhu, Haiying Xu, Sheng Yao, Gang Zhou, Yuwen Zhu, Koji Tamada, Lanqing Huang, Andrew D. Flies, Megan Broadwater, William Ruff, Jan M. A. van Deursen, Ignacio Melero, Zhou Zhu, Lieping Chen

**Affiliations:** 1 Institute of Immunotherapy, Sun Yat-Sen University, Guangzhou, Guangdong, China; 2 Department of Immunobiology and Medicine, Yale University School of Medicine, New Haven, Connecticut, United States of America; 3 Department of Oncology, Johns Hopkins University School of Medicine, Baltimore, Maryland, United States of America; 4 Department of Pediatrics, Mayo Clinic, Rochester, Minnesota, United States of America; 5 Department of Medicine, University of Navarra, Pamplona, Spain; 6 Department of Medicine, Yale University School of Medicine, New Haven, Connecticut, United States of America; University Hospital Jena, GERMANY

## Abstract

B7-H3 is a cell surface molecule in the immunoglobulin superfamily that is frequently upregulated in response to autoantigens and pathogens during host T cell immune responses. However, B7-H3's role in the differential regulation of T cell subsets remains largely unknown. Therefore, we constructed a new B7-H3 deficient mouse strain (B7-H3 KO) and evaluated the functions of B7-H3 in the regulation of Th1, Th2, and Th17 subsets in experimental autoimmune encephalomyelitis (EAE), experimental asthma, and collagen-induced arthritis (CIA); these mouse models were used to predict human immune responses in multiple sclerosis, asthma, and rheumatoid arthritis, respectively. Here, we demonstrate that B7-H3 KO mice have significantly less inflammation, decreased pathogenesis, and limited disease progression in both EAE and CIA mouse models when compared with littermates; these results were accompanied by a decrease in IFN-γ and IL-17 production. In sharp contrast, B7-H3 KO mice developed severe ovalbumin (OVA)-induced asthma with characteristic infiltrations of eosinophils in the lung, increased IL-5 and IL-13 in lavage fluid, and elevated IgE anti-OVA antibodies in the blood. Our results suggest B7-H3 has a costimulatory function on Th1/Th17 but a coinhibitory function on Th2 responses. Our studies reveal that B7-H3 could affect different T cell subsets which have important implications for regulating pathogenesis and disease progression in human autoimmune disease.

## Introduction

Upon T cell receptor-mediated recognition of MHC antigenic peptides, T cell responses to antigens, including autoreactive antigens, are orchestrated by a group of cell surface signaling molecules. These molecules could be loosely categorized into two distinct types, costimulatory or coinhibitory molecules, based on their functions in regulating T cell responses. Therefore, integrating the functional outcome of costimulatory and coinhibitory interactions determines the fate of a T cell response, which leads to response, unresponsiveness, and death [1]. Various cell surface signaling molecules have been identified and characterized, including those in the immunoglobulin (Ig) superfamily and the tumor necrosis factor (TNF) receptor and ligand superfamily. The roles of these receptors and ligands in the positive and negative control of T cell immunity and human disease, including autoimmune diseases, have been firmly established [2].

In 2001, our laboratory initially identified B7-H3 (CD276) as a costimulatory molecule that promotes an *in vitro* T cell response [3]. B7-H3 mRNA has been found in human liver, lung, bladder, testis, prostate, breast, and placenta, suggesting that B7-H3 may participate in organ-specific inflammation and autoimmune diseases. The counter-receptor for the costimulatory effect of B7-H3 was reported to be myeloid cell-like transcript 2 factor [4] whereas other study did not support this finding [5]. Similar to other B7 family homologues, B7-H3 has a single IgV- and IgC-like domain (2Ig form) with a transmembrane and intracellular tail in humans, mice, and other species. In humans, a duplicate of the classic B7-H3 (4Ig form) was also identified, but the physiological differences between the 2Ig and 4Ig form have yet to be elucidated [6–7]. The role of endogenous B7-H3 in the pathogenesis and progression of autoimmune disease has been evaluated by various laboratories using both monoclonal antibodies (mAb) and B7-H3 deficient mice (KO), but the results are somewhat contradictory with both costimulatory and coinhibitory effects being described in various model systems [8–9]. One interpretation for these contradicting results is that B7-H3 plays a differential role in the regulation of distinct T cell subsets. Therefore, the effect of B7-H3 would be determined by the dominance or bias of T cell subsets in each system or disease status. It is well known that CD4+ T cells consist of multiple functional subsets upon encountering antigens and these subsets regulate T cell responses against different antigens in different and complex environments. Th1 cells, for example, secrete IFN-γ, IL-2, and GM-CSF; they actively regulate T cell proliferation, functional maturation of CD8+ T cells, and activation of several innate immune cell components, including myeloid dendritic cells, macrophages, and granulocytes. Th1 is pathogenic in multiple human autoimmune diseases and in experimental models for EAE and CIA. Th2 cells distinguish themselves from other T cell subsets by secreting IL-4, IL-5, and IL-13; they actively promote IgE antibody production and regulate the immune response to allergens, including those involved in asthma and parasitic infections. Under the influence of IL-6, IL-21, and TGF-β, Th17 cells produces IL-17, and Th17 subsets regulate several autoimmune diseases, including EAE and CIA. The differentiation of Th cells appears to be controlled at the transcriptional level: T-bet is critical for generating Th1 cells; GATA largely regulates Th2; RORγt is a master transcription regulator for Th17 [10–12]. To delineate the role of B7-H3 in the differential regulation of T cell subsets, we constructed a B7-H3-deficient mouse strain and studied the differential roles of B7-H3 in these T cell subsets and in multiple mouse models where pathogenic T cells are biased toward specific T cell subsets.

## Methods

### Ethics statement

B7-H3 KO mice were generated in Mayo Clinic and gene targeting mice protocols were approved by the Mayo Clinic Institutional Animal Care and Use Committee. All of the experimental animals used in this study were housed in specific pathogen-free environment and performed under the protocol approved by animal facilities of Johns Hopkins University, Yale University and Sun Yat-Sen University. When mice are paralyzed in EAE induction procedure, they were provided with gel pack and/or moistened food in the cage and mice were monitored daily by both researchers and animal care staff. Animals were euthanized if they were moribund or the experiment was completed. In all cases, mice were humanely sacrificed by using carbon dioxide.

### Generation of B7-H3 KO Mice

A murine B7-H3 genomic DNA clone was identified by screening a 129/SvJ bacterial artificial chromosome (BAC) library (IncyteGenomics, St. Louis). A 4.6 kb B7-H3 upstream DNA homologous fragment and a 6.5 kb downstream DNA homologous fragment were PCR amplified from a positive BAC clone. The fragments were cloned into a pKO targeting vector that contains a neo resistant gene and a TK gene (Stratagene). The targeting vector with the B7-H3 IgV and IgC domains replaced was transfected into 129/Svj embryonic stem (ES) cells (The 129/Svj ES cell line was purchased from ATCC, SCRC-1020). Correctly targeted ES cells were confirmed by Southern blot analysis and then injected into C57BL/6 mouse blastocytes followed by implantation into foster mothers to generate chimeric mice. B7-H3 mutant mice were identified by PCR analysis of tail genomic DNA. The sequences of the PCR primers are: B7-H3 forward, 5’CGGCTCAGTCACCATCACAGGTAA; B7-H3 reverse, 5’GAGCCGCAATGAGCCTAAGGTCTA; Neo forward, 5’AGACTAGTGAGACGTGCTACTTCCA. The mice were backcrossed to a C57BL/6 or a DBA/1J background for ten generations. The same generations of homozygous or wild type mice were used for experimental analysis.

### Induction of EAE

The wild type (wt) and B7-H3 KO mice (C57BL/6, male, 8–10 week old) were immunized subcutaneously (s.c.) at both flanks with 200 μl of emulsion comprising 200 μg MOG 33–35 peptide, 400 μg *Mycobacterium Tuberculosis* (Difco) and complete Freund’s adjuvant (CFA) (Difco) on day 0; and injected intraperitoneally (i.p) with 400 μg pertussis toxin (Sigma-Aldrich) on the same day and day 2. Mice were scored every other day for signs of EAE on 0–5 scales as follows: 0, no abnormality; 1, complete loss of tail tonicity; 2, flaccid tail and abnormal gaits; 3, hind leg paralysis; 4, hind and foreleg paralysis; 5, death.

### Induction and analysis of asthma

Wt and B7-H3 KO mice (C57BL/6, male, 8–10 weeks old) were sensitized via i.p. injection of 20 μg OVA protein (Sigma-Aldrich) adsorbed to 4 mg aluminum hydroxide Gel (Sigma-Aldrich) on days 0 and 5. Then, mice were challenged with aerosolized 1% OVA in PBS using a nebulizer (Proneb Ultra II) for 60 minutes on days 12–14. Mice were sacrificed the next day (some mice were sacrificed on day 12 for *in vitro* proliferation and Th cells differentiation experiments). Blood was collected and serum was separated for OVA-specific IgE determination. The trachea was cannulated and the lungs were lavaged 3 times with a total of 1.5 ml of PBS. The bronchoalveolar lavage fluids (BALF) were centrifuged. The supernatants were collected to determine cytokine IL-5 and IL-13 levels and the cell pellets were re-suspended in 1 ml of PBS. Half a milliliter of suspended cells were counted using a Cell Viability Analyzer (Beckman Coulter) to determine the total cell counts in the BALF; the rest was spun onto microscope slides by CytoFuge and stained with Hema3 (Fisher Scientific). BALF differential cell counts were identified by counting 400 cells per slides using a high-magnification microscope. The relative numbers of each cell type were calculated by multiplying the total cell counts by the percentage of each cell type in the BALF cells. For histopathology analysis of lung inflammation, peribronchial and perivascular inflammation was evaluated on a scale of 0–4, for no inflammation (0), few inflammatory cells present (1), a few loci of inflammation (2), multiple loci of inflammation (3), inflammatory cells throughout the lung (4). The scores were presented as mean ± SEM. For mucus-secreting cells in the airways, mucous scores of 0–3 were evaluated for: no positive cells present (0), a few positive cells (1), many positive cells (2), and extensive staining of mucus-secreting cells (3)[13].

### Induction of collagen-induced arthritis (CIA)

Chicken Collagen II (CII, Sigma-Aldrich) was dissolved in 10 mM acetic acid at 4 mg/ml and emulsified with an equal volume of CFA (Difco). Wt and B7-H3 KO mice (DBA/1J, male, 8–10 week old) were immunized s.c. at both flanks with 100 μl of emulsion and a boosted with the same concentration of CII (emulsified in IFA for this immunization) 16 or 18 days after the primary immunization. Animals were evaluated by visual inspection for arthritis incidence; severity was scored individually on a 0–4 scale [14]. Scores from all four paws were added to give the total for each animal.

### *In vitro* T cell proliferation response

The brachial and axillary draining-lymph nodes (DLN) cells were isolated from wt or B7-H3 KO mice 10 days post MOG/CFA immunization. Splenocytes were removed from the OVA-immunized wt or B7-H3 KO mice 12 days after their first immunization and from wt or B7-H3 KO CIA mice 20 days after the primary immunization, respectively. The DLN cells or splenocytes were cultured in 96-well culture plates with various doses of MOG peptide, OVA, or CII proteins (heated for 30 minutes at 60°C). The proliferative activity of the T cells was assessed in triplicates by ^3^H-thymidine incorporation during the last 6 hours of the 3 days culture.

### Cytokine measurement

Cytokines in BALF and cultured supernatants were measured using OptEIA kits (BD Pharmingen) for IL-2, IL-12, IL-5, IFNγ and ELISpot Kit (R&D Systems) for IL-13. In the CIA experiments, IL-2, IL-4, IL-6, IL-10, TNF, IFN-γ, and IL-17A from the culture supernatants were assessed by CBA mouse Th1/Th2/Th17 Cytokine kit (BD Biosciences) and the data were analyzed using an FCAP Array V2.0.

### ELISA Measurement of OVA-specific IgE and anti-CII IgG

For OVA-specific IgE, ELISA plates were coated with 100 μg/ml of OVA at 4°C overnight. The plate was washed and blocked with 1% BSA for 2 h at room temperature. 1:50 diluted serum samples were incubated at room temperature for 2h, followed by treatment with HRP-conjugated anti-mouse IgE (BD Pharmingen). After washing, peroxidase substrate was added and the absorbance was read at 450 nm with an automated ELISA reader. For anti-CII IgG, serum was collected from CIA mice 20 days after their primary immunization and the levels of anti-type II collagen Ab (IgG) were assayed using a mouse anti-type II collagen IgG assay kit (Chondrex Inc) according to manufacturer's instructions.

### *In vitro* differentiation of Th1 and Th2 cells and intracellular staining

DLN cells (EAE model) or splenocytes (asthma model) from wt or B7-H3 KO mice were cultured with MOG peptide (40 μg/ml) or OVA (50 μg/ml) under Th1 (10 ng/ml IL-12, 10 μg/ml anti-IL-4) or Th2 (10 ng/ml IL-4, 10 μg/ml anti-IFNγ and 10 μg/ml anti-IL-12) polarizing conditions for 5 days. 1 μg/ml of anti-CD3 and 1 μl/ml of GolgiPlug was added during the last 6 h. The cells were stained for surface CD4, then fixed and permeabilized using the Cytofix/Cytoperm kit (BD Biosciences) followed by intracellular staining for IFN-γ and IL-4.

### RT-PCR and Real-time quantitative RT-PCR (qRT-PCR) assay

Total RNA was extracted using the RNeasy kit (Qiagen). First-strand cDNAs was synthesized and amplified by RT-PCR using a primer set for mouse B7-H3: 5’ GACACGGATGCCACCCTACGCTG (forward) and 5’CTGTGATGGTGACTGAGCCGTGAG (reverse). β-actin mRNA levels were determined as a control for each sample. For real-time qRT-PCR, total RNA was extracted using RNeasy (Qiagen) and was reverse transcribed to cDNA using Taqman reverse transcription reagents (Applied Biosystems) according to the manufacturer’s instructions. Primer and probe sets were obtained from Applied Biosystems. Real-time qPCR was performed using the Taqman Universal PCR Master Mix and an Applied Biosystems 7500 Fast Real-Time PCR system. Relative gene expression levels were normalized to the 18S rRNA endogenous control levels.

### Histopathology and Immunohistochemistry

Lungs from allergic mice were fixed with Streck Tissue Fixative (Streck). The lung sections were stained with hematoxylin and eosin (H&E) or Periodic Acid-Schiff (PAS) (Sigma-Aldrich) for histological evaluation. Intact spinal cords were flushed out by hydrostatic pressure from mice 20 days after MOG/CFA immunization and fixed in 10% formalin. The sections were stained with H&E to assess inflammatory lesions. The hind paws from normal or CIA mice were collected 40 days after their primary immunization. The skins were removed from their paws and the bones were decalcified using Formical 2000 (Decal Chemical Corporation) for 24 hrs followed by fixation with SafeFix II (Fisher Scientific). After deparaffinization and rehydration, the tissue sections were stained with H&E for histological analysis. For immunohistochemistry, endogenous peroxidases, nonspecific binding sites and endogenous avidin/biotin were blocked using 0.3% H_2_O_2_, Block AEC (AbD Serotec), and Avidin Blocking Buffer (Vector Laboratories), respectively. Then, sections were incubated with an anti-mouse biotin-conjugated B7-H3 mAb (5 μg/ml) at 4°C overnight. Subsequently, the Catalyzed Signal Amplification System (DAKO, K1500) was used for staining. The slides were counterstained with hematoxylin. Monoclonal antibody to murine B7-H3 (clone mB7-H3.7H) was generated by immunizing an Armenia hamster with mouse B7-H3Ig recombinant fusion protein, as described previously [3].

### Statistical analysis

All statistics were performed using a two-tailed Student’s *t* test. All bars are shown as mean ± standard error of the mean (SEM).

## Results

### Generating B7-H3 knockout (KO) mice

**Fig 1:**
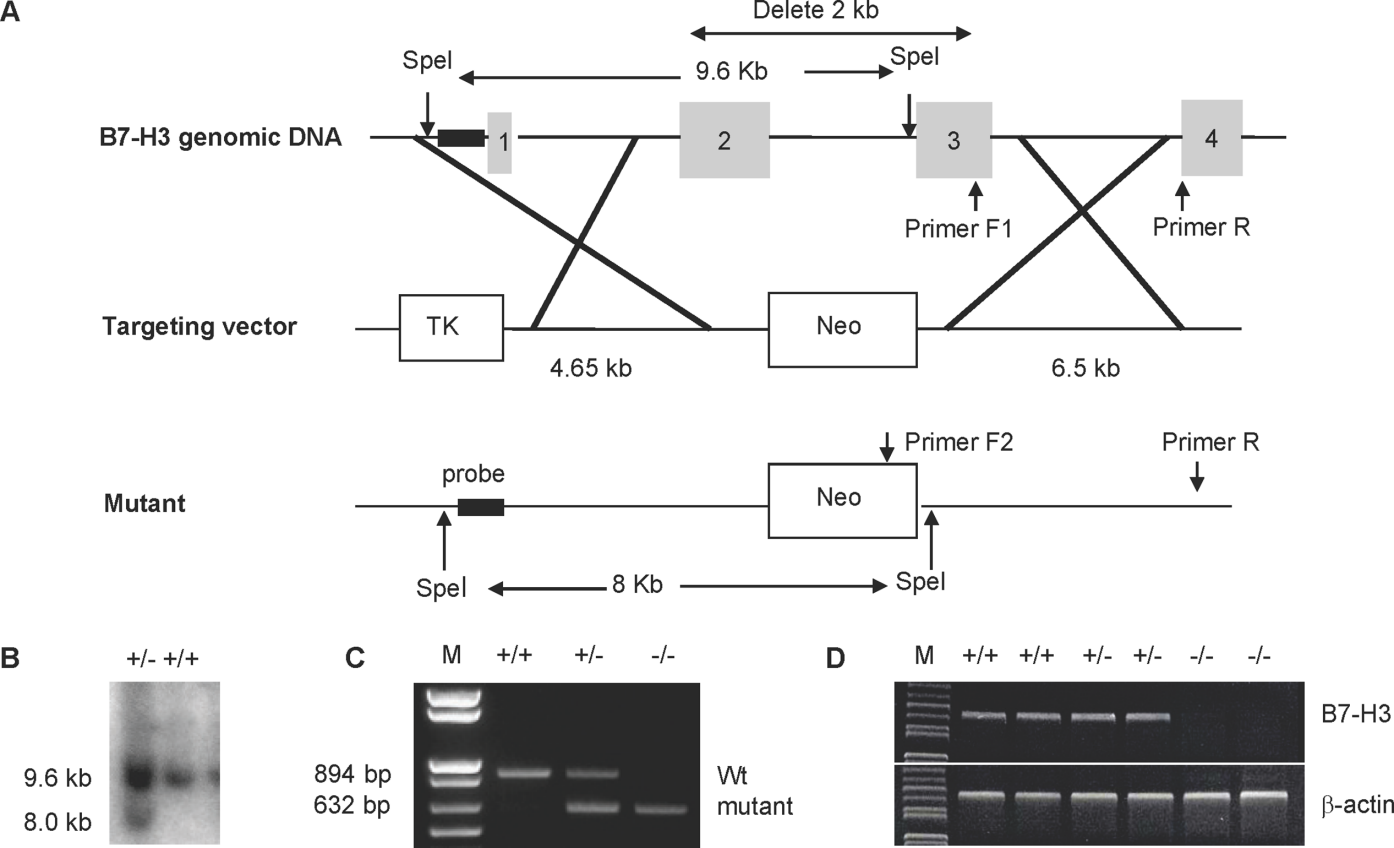
Generation and characterization of B7-H3 KO mice. (A) Mapping of the B7-H3 genomic locus, targeting vector, and the replaced allele. (B) Southern blot analysis of SpeI-digested DNA from targeted embryonic stem (ES) cell clones. The wild-type allele generated a 9.6-kb fragment, and the targeted allele yielded an 8.0-kb fragment. (C) PCR identification of genomic DNA isolated from tails of wild type (+/+), heterozygous (+/−) or homozygous (−/−) B7-H3 mutant mice. The wild type allele generated an 894-bp fragment and the targeted allele generated a 632-bp fragment. (D) Examination of B7-H3 mRNA by RT-PCR. The cDNA was generated from the spleens of wild type (+/+), heterozygous (+/−) or homozygous (−/−) B7-H3 mutant mice using primers specific for B7-H3 or β-actin (control).

The B7-H3 KO mice were generated using homologous recombination in embryonic stem (ES) cells. The targeting vector was constructed to replace exon 2 and exon 3 of the B7-H3 gene with a TK gene and a neomycin resistance gene. Homologous recombination by this targeting vector in ES cells deleted a 2 kb region that encodes for the IgV and IgC domains of B7-H3 (Fig 1A). Using Southern blot analysis, we identified one correctly targeted ES clone from the 480 G418-resistant colonies (Fig 1B), and then injected this clone into 129 mice blastocytes to generate chimeric mice. The strains of C57BL/6 genetic background mice were established by backcrossing chimeric mice with C57BL/6 mice for >10 generations. Wild type, heterozygous, or homozygous B7-H3 mutant mice were identified using mouse tail genomic DNA and PCR analysis (Fig 1C). The absence of B7-H3 mRNA in KO mice was also confirmed by RT-PCR using cDNA generated from spleen cells (Fig 1D). The mutant mice were monitored up to one year and were viable, fertile, normal in size, and did not display any visible physical abnormalities. In addition, the mutant mice displayed normal numbers and ratios of T, B, NK, and DC cells in the thymus, spleen, and lymph nodes (data not shown).

### B7-H3 KO mice are resistant to EAE

**Fig 2:**
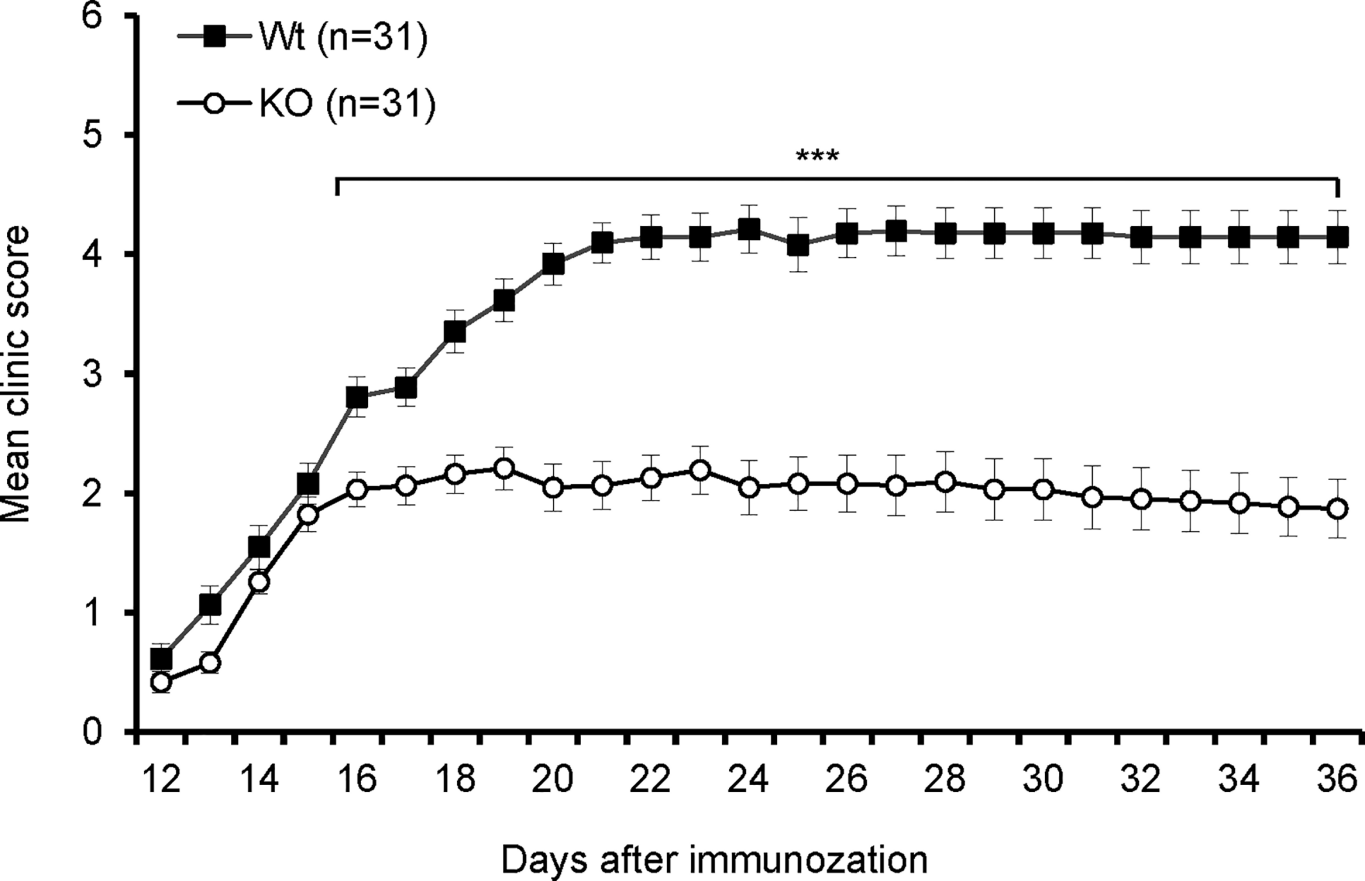
B7-H3 KO mice are resistant to EAE. EAE incidence and severity in wild type and B7-H3 KO mice. Data are presented as an average clinical score from four independent experiments (5–10 mice each group). There were significant differences between two groups starting on day 16. Data are shown as mean ± SEM,*p<0.05, **p<0.01, ***p<0.001, versus control.

To investigate the *in vivo* function of B7-H3 in autoimmune disease, we first examined the role of B7-H3 on the development of EAE. As shown in Fig 2, the onset of disease in B7-H3 KO mice was similar to that in wt mice; however, B7-H3 KO mice exhibited attenuated symptoms during the course of EAE development, specifically 16 days post-immunization. The mean maximal clinical score in B7-H3 KO mice was markedly less than that in wt mice. In most B7-H3 KO mice, the disease was not fatal and the mice demonstrated a partial recovery from their EAE symptoms (Fig 2). Consistent with these observations, histopathological examinations showed a massive infiltration of mononuclear cells in the proximal spinal cord region of wt mice, while there were fewer inflammatory cells in the same region of B7-H3 KO mice (S1 Fig). Though disease severity was significantly reduced in B7-H3 KO mice, their disease incidence is also 100%, the same as wt group. These results indicate that our newly developed B7-H3 KO mice are more resistant to EAE than wt mice.

### Myelin-specific T cell response is reduced in B7-H3 KO mice

**Fig 3:**
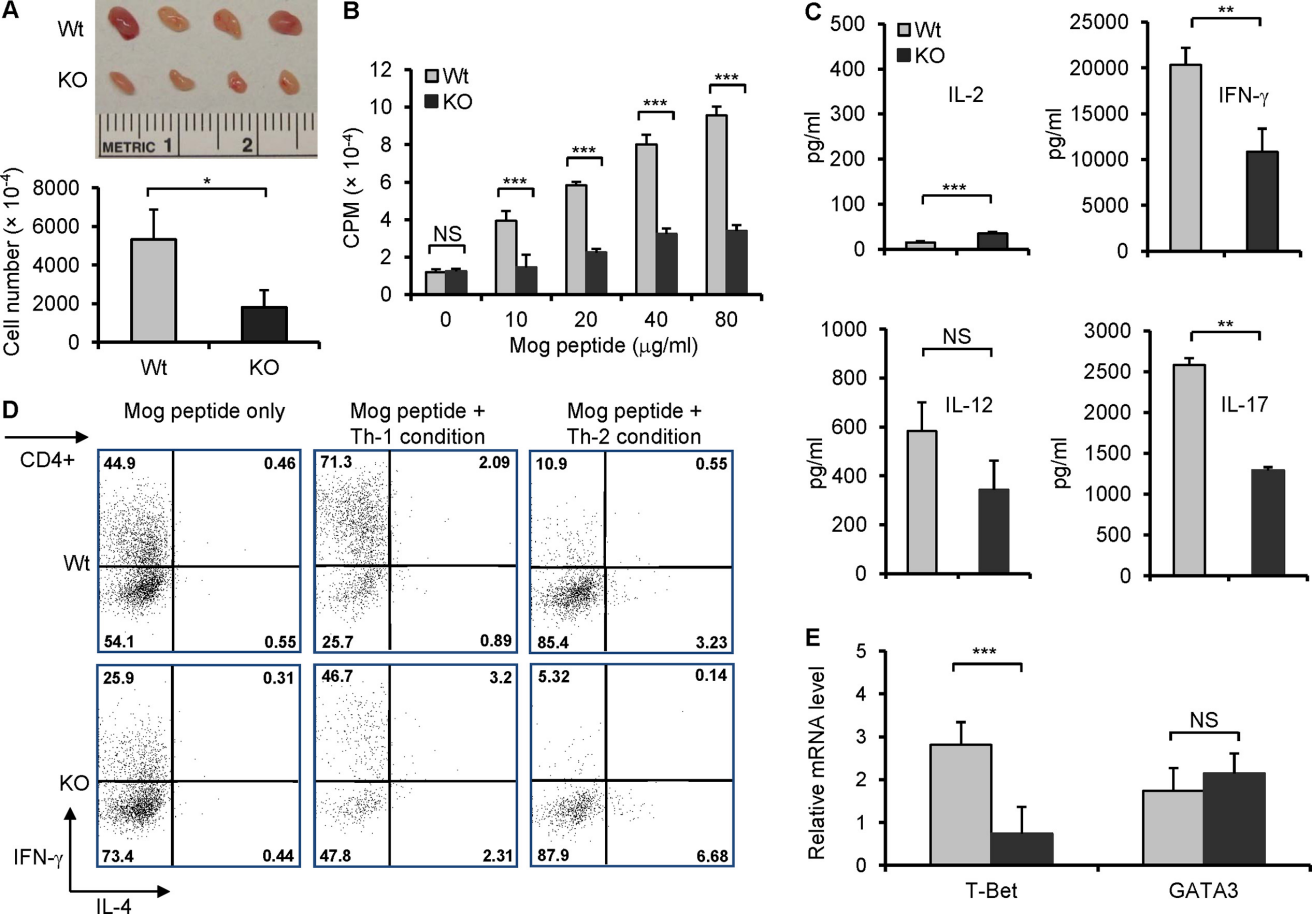
Decreased MOG-specific T cell responses in B7-H3 KO mice. The brachial and axillary draining-lymph nodes (DLN) were removed from wild type and B7-H3 KO mice 10 days after MOG/CFA immunization. (A) Comparing DLN size and number. Representative image of wt and B7-H3 KO mice DLNs (top), which taken from one mouse per group. DLNs number counting is from five mice per group (bottom). Data are representative of three independent experiments. (B) Proliferative response of DLN cells to MOG peptide, as determined by incorporation of ^3^H-TdR. DLNs were collected from five mice per group. Data are representative of four independent experiments with similar results. (C) Cytokine IL-2, IL-12, IL-17, and IFN-γ concentrations in cultured supernatants of DLN cells were measured by ELISA 2 days after restimulation with MOG peptide (40 μg/ml). Data are representative of three independent experiments with similar results. (D) DLN cells (mixed 5 individual cells per group) were cultured with MOG peptide (40 μg/ml) for 5 days under Th1 or Th2 polarizing conditions. Then, Th1 and Th2 cell subpopulations were analyzed by intracellular cytokine staining. Data are representative of 3 independent experiments with similar results. (E) Real-time PCR analysis of T-bet and GATA3 were performed on cDNA from wt and B7-H3 KO mice DLN cells, 5 individual cells per group in triplicates. Data are representative of 2 independent experiments. Data are shown as mean ± SEM, *p<0.05, **p<0.01, ***p<0.001, versus control.

Myelin-specific T cells play an important role in EAE animal models. To confirm our *in vivo* findings, we investigated whether there was a difference between wt and B7-H3 KO mouse T cell-specific responses to MOG 33–35 peptide. The brachial and axillary draining-lymph nodes (DLN) were removed from wt or B7-H3 KO mice 10 days after MOG/CFA immunization. Although the DLNs from both the wt and B7-H3 KO mice exhibited swelling, the latter was visibly smaller. The total cell number obtained from DLN in the B7-H3 KO mice was half the number or less of wt mice (Fig 3A). The DLN cells were then isolated and re-stimulated with various doses of MOG peptide *in vitro*. DLN cells isolated from wt mice induced a strong proliferative response upon MOG peptide stimulation and in a dose-dependent manner; whereas DLN cells isolated from B7-H3 KO mice responded poorly (Fig 3B). Meanwhile, we determined the concentrations of T-helper cell type 1 (Th1) cytokines and IL-17 in the culture supernatants. The DLN cells from B7-H3 KO mice produced fewer IFN-γ, IL-12, and IL-17 than the DLNs isolated from wt mice (Fig 3C). We further assessed the Th1/Th2 differentiation of DLN cells from primed wt or B7-H3 KO mice *in vitro*. In the presence of MOG peptides, the DLN cells from the wt mice produced a significant number of IFN-γ producing cells under Th1 polarizing conditions, but very few numbers of IL-4 producing cells under Th2 polarizing conditions; this is consistent with EAE's Th1-type response. However, DLN cells from B7-H3 KO mice generated a decreased proportion of IFN-γ producing cells under Th1 polarizing conditions (Fig 3D). T-bet has been identified as a key transcription factor in Th1 cell development, so we further compared the levels of T-bet mRNA in the DLN cells from wt and B7-H3 KO mice 10 days after MOG/CFA immunization using real time quantitative RT-PCR. T-bet mRNA levels were reduced in DLN cells from the B7-H3 KO mice compared with that of wt mice. In contrast, there was not a difference between the levels of GATA-3 mRNA in wt and B7-H3 KO mice (Fig 3E). Therefore, the reduced myelin-specific T cell responses to MOG peptide which may be caused by the decreased Th1 cell differentiation, proliferation and/or survival.

### Enhanced airway allergic response in B7-H3 KO mice

**Fig 4:**
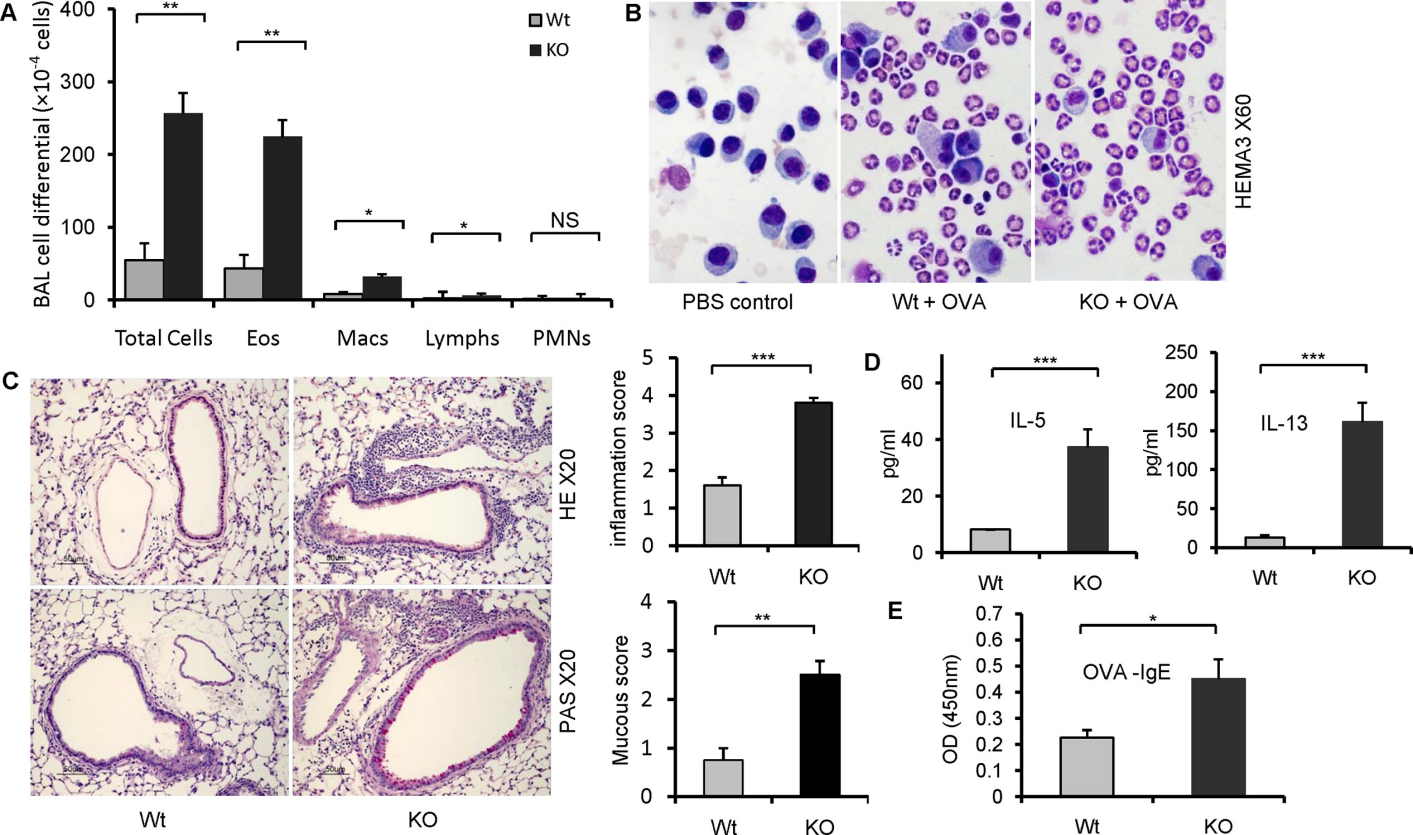
Enhanced airway allergic responses in B7-H3 KO mice. (A) Total and eosinophil cell counts in BALF from OVA protein-immunized and challenged wild type or B7-H3 KO mice. Data were from 5 individual mice per group each experiment and data are representative of four independent experiments. Eos, eosinophils; Macs, macrophage; Lymphs, lymphocytes; PMNs, polymorphonuclear neutrophils. (B) Characterization of inflammatory cells in BALF. The cells were spun to slides, and then stained with HEMA3 (Original magnification: ×60). (C) Lung paraffin sections of naïve, allergic wild type, and B7-H3 KO mice were stained with H & E and PAS (Original magnification: ×20). Peribronchial and perivas cular inflammation and mucus-secreting cells in the airways were scored (right plots). (D) Th2 cytokine levels in BALF of wild type or B7-H3 KO mice assessed by ELISA. (E) Levels of serum OVA-specific IgE were determined by ELISA in wild type and B7-H3 KO mice after OVA protein challenge. Data are shown as mean ± SEM, *p<0.05, **p<0.01, ***p<0.001, versus control.

Next, we examined whether a reduction in B7-H3 could cause an imbalance in the Th1 and Th2 response in Th2 lymphocytes' predominant immune response. Wt and B7-H3 KO mice were immunized via i.p. injection of OVA-aluminum, and then challenged with aerosolized OVA protein. The bronchoalveolar lavage fluids (BALF) were harvested and the total and differential BALF cell counts were determined. The total BALF cell counts in OVA-immunized and challenged B7-H3 KO mice were significantly higher than in wt mice; this increase was mainly observed in eosinophils (Fig 4A and 4B). Histological examination of lung tissue revealed numerous inflammatory cells concentrated around the bronchovascular bundles in B7-H3 KO mice, and many PAS-positive cells were observed in small airways. Quantitation of these focal inflammation and mucus production demonstrate a significantly increased inflammation and mucus production scores. Meanwhile, there was limited infiltration and only a few PAS-positive cells in the same regions in wt mice (Fig 4C). This indicates that airway inflammation is more serious in the lung tissue of B7-H3 KO mice. Analysis of the Th2 cytokines, IL-5 and IL-13, in BALF showed that B7-H3 KO mice exhibited increased levels of IL-5 and IL-13 in BALF compared with wt mice (Fig 4D). We also assayed OVA-specific IgE levels in serum from wt and B7-H3 KO mice that were immunized and challenged with OVA and found that OVA-specific IgE was increased in B7-H3 KO mice compared with wt mice (Fig 4E). These results indicate that B7-H3 KO mice enhance Th2-mediated allergic responses in the airway.

### Elevated Th2 response in B7-H3 KO

**Fig 5:**
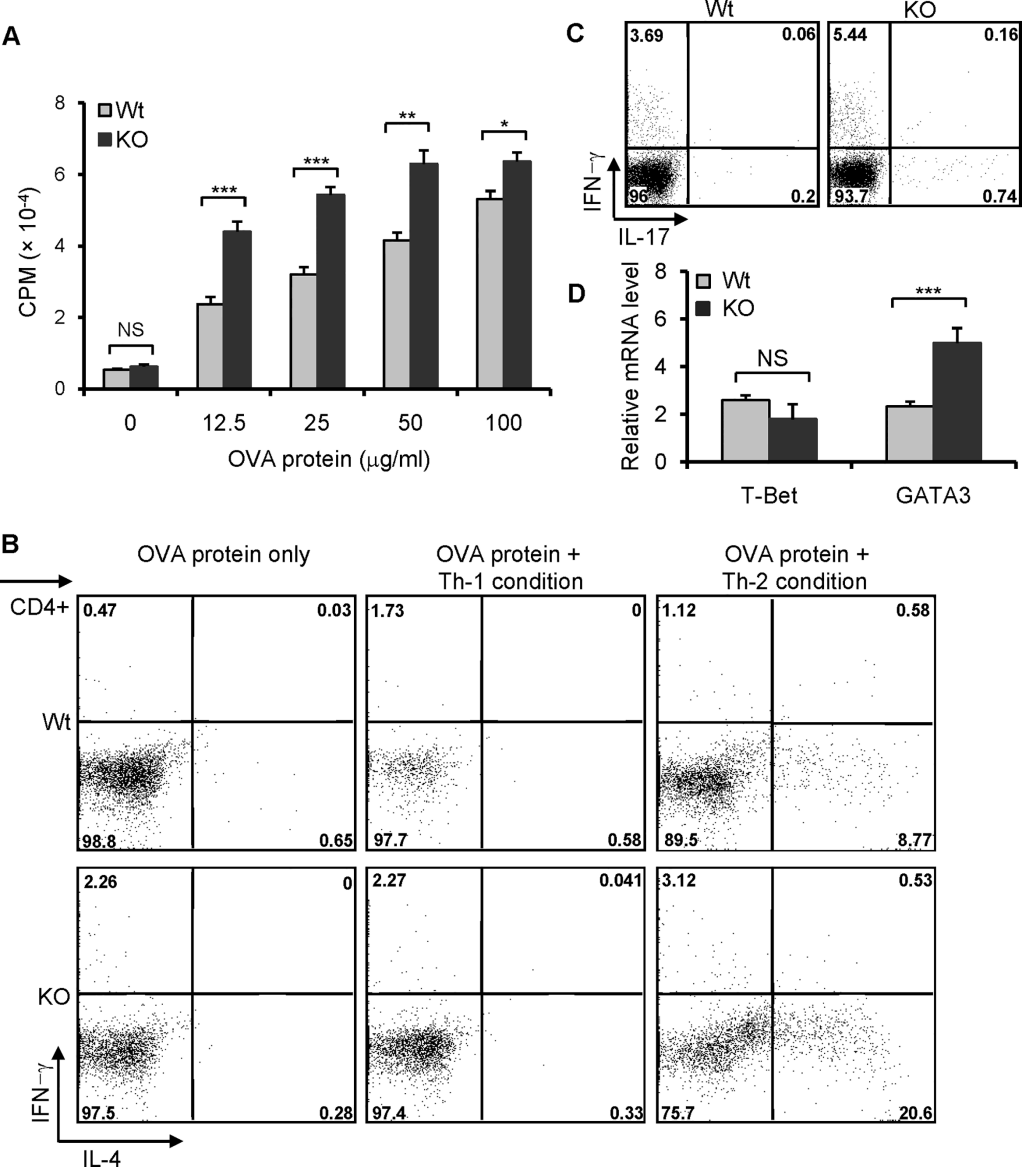
Predominant Th2 response in B7-H3 KO mice. Spleens were removed from OVA protein-immunized wild type and B7-H3 KO mice on day 12. (A) Splenocytes were restimulated with varying concentrations OVA protein. Cell proliferation was determined by ^3^H-TdR incorporation. 5 mice per group and data are representative of 3 independent experiments with similar results. (B) Splenocytes (mixed 5 individual cells per group) were cultured with OVA protein (50 μg/ml) for 5 days under Th-1 or Th-2 polarizing conditions. Th1 and Th2 cell subpopulations were analyzed by intracellular cytokine staining. Data are representative of three independent experiments with similar results. (C) Splenocytes from wt or B7-H3 KO mice were restimulated with PMA (5ng/ml) + ionomycin (250 ng/ml) and GolgiPlug (1 μl/ml) *in vitro* for 5 hrs. IL-17 producing cells were analyzed by flow cytometry. 5 mice per group and data are representative of 2 independent experiments. (D) Real-time PCR analysis of T-bet and GATA3 were performed on the cDNA from 5 individual splenocytes per group in triplicates. Data are representative of 2 independent experiments. Data are shown as mean ± SEM, *p<0.05, **p<0.01, ***p<0.001, versus control.

To confirm further that the increased airway inflammation in the lungs of B7-H3 KO mice is associated with an enhanced Th2 response, the splenocytes from OVA-immunized wt or B7-H3 KO mice were re-challenged with OVA protein *in vitro*. Splenocytes from B7-H3 KO mice exhibited a marked proliferation compared to wt mice (Fig 5A). When stimulated with OVA protein under Th2 polarizing conditions, splenocytes from B7-H3 KO mice induced a larger proportion of IL-4 producing cells than that from wt mice (Fig 5B). Therefore, the enhanced Th2 cell polarization in B7-H3 KO mice involved a more severe allergic response in the airways. We also assessed the production of IL-17 in splenocytes from OVA protein challenged wild type or B7-H3 KO mice following restimulation *in vitro* with PMA + ionomycin for 5 hrs. Intracellular cytokine staining revealed that there are fewer IL-17+ cells in CD4+ splenocytes for both wt and B7-H3 KO mice (Fig 5C). Although the number of IL-17 producing cells in B7-H3 KO mice is a little higher than wt mice, it is uncertain whether IL-17 is involved in the mechanism by which B7-H3 KO mice develop more severe airway inflammation. To further understand the mechanism whereby Th2 response is enhanced in B7-H3 KO mice, we analyzed the expression of GATA3, a principal transcriptional activator of Th2 cell differentiation, in the splenocytes of wt and B7-H3 KO mice with induced asthma. Real time RT-PCR analysis indicated that GATA3 mRNA levels were significantly increased in B7-H3 KO mice compared with that in wt mice (Fig 5D). These results suggest that enhanced GATA3 mRNA expression in CD4+ cells from B7-H3 KO mice may be associated with the predominant Th2 cell responses during the development of allergic respiratory inflammation.

### Decreased CIA severity in B7-H3 KO mice

**Fig 6:**
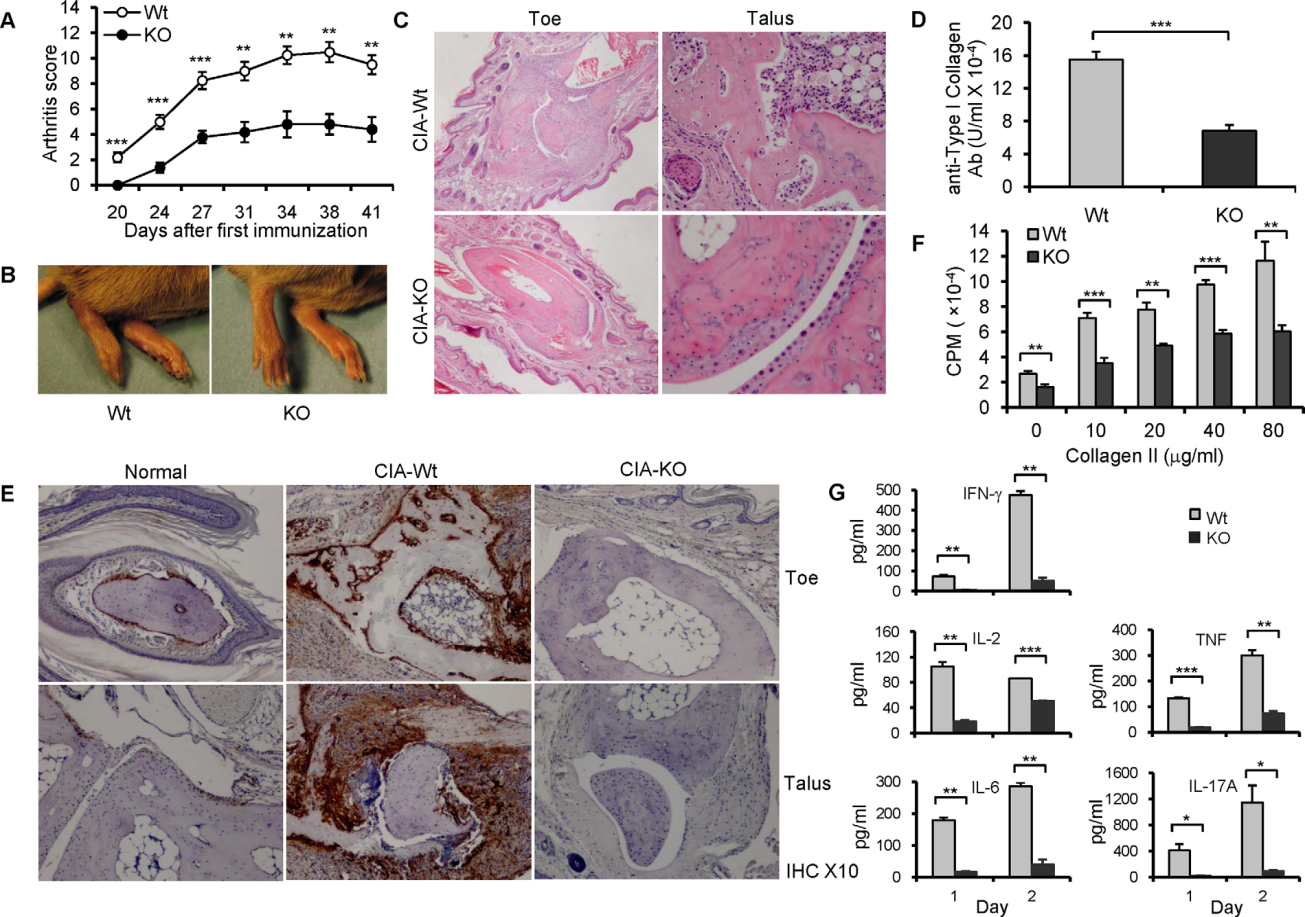
B7-H3 KO mice are resistant to CIA. (A) The arthritis score in CIA mice. 5–10 mice per group per experiment and data shown are representative of 3 independent experiments with similar results. (B) Representative images of hind paws from wild type (left) and B7-H3 KO (right) CIA mice on 40 days after primary immunization. (C) Representative hematoxylin and eosin—stained sections of toe and talus joints (original magnification ×4 and ×10, respectively) from wild type and B7-H3 KO CIA mice. (D) Serum anti-type II collagen Ab (IgG) levels (100 units is approximately 0.1 mg IgG antibody/ml). Data shown are representative of 3 independent experiments. (E) Staining for B7-H3 in normal and arthritic joints. Hind toe joints from normal, CIA-wild type, and B7-H3 KO mice (Original magnification ×10). (F) Splenocytes from wild type and B7-H3 KO CIA mice were restimulated with chicken CII protein in various concentrations. 5 mice per group each experiment. Cell proliferation was determined by ^3^H-TdR incorporation. Data shown are representative of 3 independent experiments. (G) Th1/Th2/Th17 Cytokine concentrations of cultured supernatants from chicken CII protein (20 μg/ml)-restimulated splenocytes (mixed 5 individual cells per group) after 1 and 2 days were assessed using mouse Th1/Th2/Th17 Cytokine kits (CBA); data were analyzed using FCAP Array V2.0. Data shown are representative of two independent experiments. Data are shown as mean ± SEM, *p<0.05, **p<0.01, ***p<0.001, versus control.

CIA is the most widely used mouse model for rheumatoid arthritis, a common autoimmune disease. The CIA model is thought to be an auto-reactive T and B cell-dependent arthritis, and recent studies have suggested that Th17 cells play a central role in the pathogenesis of CIA. Therefore, we tested the effect of B7-H3 in the development of CIA. B7-H3 KO mice exhibited a later onset, an attenuated severity of arthritis based on the assigned arthritis score (Fig 6A), and a decreased severity in joint swelling (Fig 6B) compared with wt mice. Clinical observation was further verified by histological examination. H&E staining of arthritic paw joints from wt mice revealed severe synovial hyperplasia, infiltration of inflammatory cells, and destructive cartilage and bone. In contrast, the levels of inflammation, infiltration, and cartilage erosion were markedly alleviated in B7-H3 KO mice (Fig 6C). In addition, CII-specific IgG levels in sera from B7-H3 KO mice were significantly lower than those from wild type mice, as determined by ELISA 20 days after primary immunization (Fig 6D). Our results indicate that B7-H3 KO mice have significantly decreased symptoms of CIA.

### B7-H3 expression is correlated with arthritic inflammation

Next, we investigated whether arthritic inflammation was associated with local B7-H3 expression. Mouse paw joint sections in normal mice were immunohistochemically stained for B7-H3; the osteoblasts surrounding the calcaneus were positive for B7-H3, as well the sections of the distal tibia. Staining sections of wt mice with CIA showed a heightened expression of B7-H3 that was accompanied by an increase in osteoblast number, compared with normal tissue. This effect was completely lost in CIA-B7-H3 KO mice, which demonstrated a complete lack of B7-H3 expression in osteoblasts (Fig 6E). Therefore, B7-H3 is induced during arthritic inflammation of paw joints, which is positively correlated with the progression of arthritic diseases.

#### CII specific T cell proliferative response and cytokine production are reduced in B7-H3 KO mice

To investigate CII antigen-specific T cell response and cytokine production in CIA wild type and B7-H3 KO mice, splenocytes were removed from arthritic mice 20 days after the primary immunization and were restimulated with different doses of CII protein *in vitro*. Splenocyte response to CII was significantly attenuated in the B7-H3 KO mice compare with that in wt mice (Fig 6F). Accordingly, cytokine IFN-γ, IL-2, IL-6, TNF, and IL-17 production was significantly reduced in cultured splenocytes from B7-H3 KO mice (Fig 6G) while IL-4 and IL-10 were expressed at minimal levels (<10pg/ml) (data not shown). These results suggest that Th1, Th17, and B cell responses were suppressed in the CIA-B7-H3 KO mice.

## Discussion

Here, we construct a new B7-H3 deficient mouse strain and use this new strain to test how B7-H3 regulates the immune response to antigen in mouse models of autoimmune disease and asthma. We reveal an unexpected role for B7-H3: B7-H3 differentially regulates Th1/Th17 and Th2 responses in the models we studied. A lack of B7-H3 ameliorated the effects of EAE and significantly decreased symptoms of CIA, which were accompanied by a decrease in Th1- and/or Th17-type cytokine responses to autoantigens. In contrast, we also noted a significantly enhanced Th2-type cytokine response and increased airway inflammation in B7-H3 KO mice. Thus, our findings not only reveal a dichotomy for B7-H3's role in the regulation of T cell subsets, but also provide important clues that will assist in designing new immune modulations that target this pathway and treat disease.

The effect of B7-H3 on the regulation of T cell responses has been evaluated in *in vitro* cell culture systems and various murine models using transfection to overexpress B7-H3 monoclonal antibodies (mAb) against B7-H3 and B7-H3 KO mice. These studies resulted in inconsistent and conflicting results from different laboratories. *In vitro* studies using human recombinant B7-H3 showed enhanced T cell growth in the presence of TCR engagement, which led to the hypothesis that B7-H3 is a costimulatory molecule for T cell growth [3]. Using transfection to overexpress B7-H3 on cancer cells, researchers enhanced immune responses, especially in CD8+ T cell compartments; this was demonstrated by several investigators [15–18]. Therefore, B7-H3 over-expression seemed costimulatory in T cell responses. Various antibodies against murine B7-H3 were generated independently by different laboratories and have been evaluated in mouse models and in *in vitro* systems; these results are inconsistent. For example, infusion of a B7-H3 mAb accelerated the progression of diseases with an enhanced Th1 T cell response in a MOG peptide-induced EAE model [19]. In addition, a B7-H3 mAb enhanced the Th2-mediated T cell response during induction of experimental allergic conjunctivitis [20]. While these data support a role for endogenously expressed B7-H3 to suppress CD4+ T cell responses, several studies employing different antibodies suggest a possible role for B7-H3 in the promotion of Th2 and Th1/CD8 T cell responses. Administration of a B7-H3 mAb reduced airway hypersensitivity by suppressing Th2 cytokine production and decreasing the number of eosinophils in the airway [21]. Furthermore, an independently generated B7-H3 mAb suppressed a CD8+ and CD4+ T cell-mediated contact hypersensitivity [4]. Although these mAbs all claimed to be “blocking or antagonist” antibodies, it is unknown whether these mAbs are truly antagonistic or could behave as both an antagonist and agonist. Because B7-H3's counter-receptor has yet to be characterized, a simple and straightforward blocking assay is not yet available. In addition to serving as ligands, several B7 family molecules including B7-2 and B7-H1 could also serve as receptors [2]. Therefore, it remains possible that B7-H3 could serve as a receptor and some of these B7-H3 mAbs could signal via B7-H3, as suggested in a recent study [22].

Two B7-H3 KO mouse strains were independently generated and characterized in addition to our current study [23–24]. Wang and colleagues showed that survival of allogeneic islets and cardiac grafts were significantly prolonged, accompanied by a decreased T cell response in B7-H3 KO mice. While this finding supports a costimulatory function for B7-H3 in T cell responses, the effect of B7-H3 on T cell subsets and their contribution to autoimmune disease were not evaluated [23]. In an independently generated B7-H3 KO strain, Suh and colleagues showed a small but significant increase in Th1-mediated lung inflammation, whereas Th2 responses remained unchanged in a cytokine/aerosolized ovalbumin-induced lung inflammation model [24]. Similarly, this strain showed a small increase in clinical score, autoantibody production, and a slightly earlier onset in MOG-induced EAE models. In this model, CTL responses to LCMV were indifferent. Due to the relatively weaker phenotypes across all of the tests in this mouse strain, a different genetic background (F2 C57BL/6 x 129/Ola or F6 BALB/c) and a different lung model of inflammation, a direct comparison between this model and our strain is difficult. In a lung inflammation model, Suh *et al* used GM-CSF with or without IL-12 to prime the lung before using OVA challenge to induce the Th1 or Th2-biased status. In our lung inflammation/asthma model, the B7-H3 KO strain was challenged with OVA in alum adjuvant to bias Th2 responses. Therefore, a significantly enhanced Th2 response was observed in our studies, where indifference was observed in the model produced by Suh and colleagues. In a MOG-peptide induced EAE model, a small increase in the clinical score and accumulation of autoantibodies were observed [24]. These results suggest a role for B7-H3 in suppressing Th1 function since the EAE in this model is mainly mediated by Th1 and Th17. In our study, a similar method was used to induce EAE but we observed a dramatic decrease in the clinical score, infiltration of lymphocytes into CNS, T cell response to MOG antigen, Th1 and Th17 cytokine, and T-bet mRNA in B7-H3 KO mice (Fig 3). Thus, our results indicate a positive regulatory role of B7-H3 in EAE. One possible reason for the difference between these two models may be the genetic background: genetic variations in F2 C57BL/6 x 129/Ola mice may be larger than in our strain of C57BL/6 [24].

It is particularly interesting that B7-H3 KO mice were resistant to CIA induction, showed fewer clinical symptoms and a decreased pathogenesis, including diminished infiltration of inflammatory cells to the joints, decreased T cell and antibody responses to collagen, and fewer inflammatory cytokines including those secreted by Th1 and Th17. Although B7-H3 mRNA is widely distributed in various normal tissues, the protein is not typically detectable. In previous mouse studies, B7-H3 was found expressed on osteoblasts which required a late phase of osteoblast differentiation [25]. Using immunohistochemical staining with anti-B7-H3 mAb, we demonstrated that B7-H3 cell surface proteins could be detected, albeit in low levels, in the paw joints of normal mice, mainly expressed in osteoblasts on the surface cartilage. Importantly, the expression of B7-H3 is drastically upregulated in collagen-immunized mice and was correlated with the level of arthritic inflammation and bone destruction. The clinical symptoms of CIA and joint inflammation and destruction were markedly reduced in B7-H3 KO mice compare to the control mice. *In vitro* experiments demonstrated that collagen-specific T cell responses were significantly decreased and IFN-γ, TNF and IL-17 cytokine production was accordingly reduced in B7-H3 KO mice. Our observations suggest that B7-H3 may be an important mediator of pathogenesis and progression in inflammatory arthritis.

Taken together, our results support the notion that B7-H3 differentially regulates T cell subsets by costimulating Th1 and Th17 while suppressing Th2 responses. This finding may explain, at least in part, the previously contradictory findings in various model systems. The molecular basis for this differential effect, however, has yet to be characterized. This will largely rely on the discovery of the B7-H3 counter-receptor and, in this regard, a different counter-receptor on these T cell subsets may cause the observed effects. Finally, our findings have important implications for the manipulation of B7-H3 in clinical applications to treat human disease.

## Supporting Information

S1 Fig Representative images of lumbar spinal cord sections from wild type and B7-H3 KO mice on day 20 post-immunization. The paraffin tissue sections were stained with hematoxylin and eosin (original magnification: ×5 and ×20).
(TIF)
