# Supplementary figures and images for: B7-H3 Promotes Pathogenesis of Autoimmune Disease and Inflammation by Regulating the Activity of Different T Cell Subsets
Source: PLoS One. 2015 Jun 11;10(6):e0130126. doi: 10.1371/journal.pone.0130126 (PMC4465912; doi:10.1371/journal.pone.0130126)

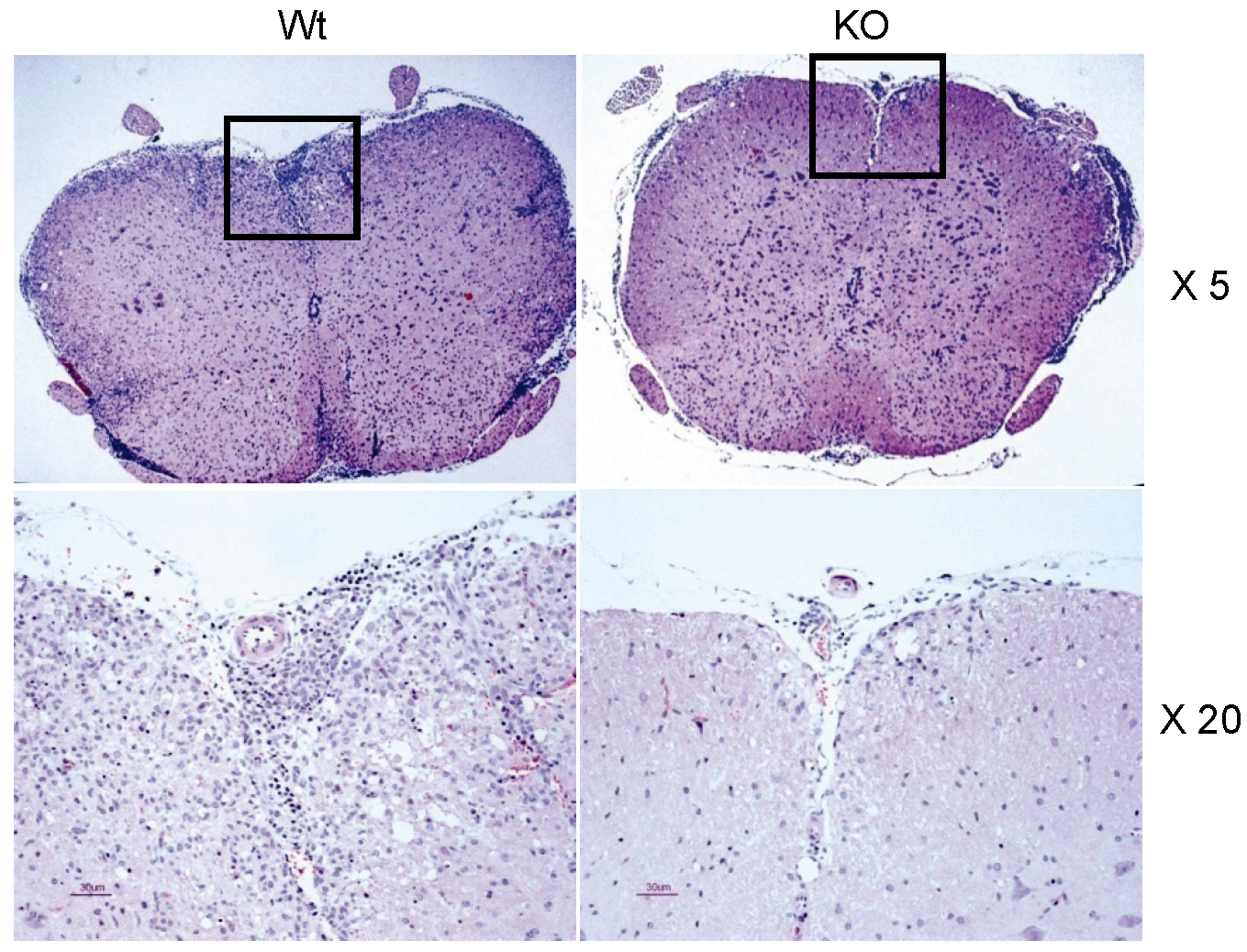

Supplement: S1 Fig — The paraffin tissue sections were stained with hematoxylin and eosin (original magnification: ×5 and ×20). (TIF) [file pone.0130126.s001.tif]
